# Defining the external implementation context: an integrative systematic literature review

**DOI:** 10.1186/s12913-018-3046-5

**Published:** 2018-03-27

**Authors:** Dennis P. Watson, Erin L. Adams, Sarah Shue, Heather Coates, Alan McGuire, Jeremy Chesher, Joanna Jackson, Ogbonnaya I. Omenka

**Affiliations:** 1 Department of Social and Behavioral Sciences, Indiana University Richard M. Fairbanks School of Public Health, 1050 Wishard Blvd, Indianapolis, IN 46202 USA; 2 Department of Psychology, Indiana University Purdue University-Indianapolis, 420 N Blackford St, Indianapolis, IN 46202 USA; 3 Indiana University-Purdue University Indianapolis, School of Health and Rehabilitation Sciences, 1050 Wishard Blvd, Indianapolis, IN 46202 USA; 4 Indiana University-Purdue University Indianapolis, University Library, Center for Digital Scholarship, 755 W. Michigan St, Indianapolis, IN 46202 USA; 5 Richard L. Roudebush VA, 1481 W. 10th St, Indianapolis, IN 46202 USA; 6 Department of Environmental Health Sciences, Indiana University Richard M. Fairbanks School of Public Health, 1050 Wishard Blvd, Indianapolis, IN 46202 USA; 7 Department of Health Policy and Management, Indiana University Richard M. Fairbanks School of Public Health, 1050 Wishard Blvd, Indianapolis, IN 46202 USA

**Keywords:** Implementation context, External context, Local context, Outer setting, Integrative review, Systematic review

## Abstract

**Background:**

Proper implementation of evidence-based interventions is necessary for their full impact to be realized. However, the majority of research to date has overlooked facilitators and barriers existing outside the boundaries of the implementing organization(s). Better understanding and measurement of the external implementation context would be particularly beneficial in light of complex health interventions that extend into and interact with the larger environment they are embedded within. We conducted a integrative systematic literature review to identify external context constructs likely to impact implementation of complex evidence-based interventions.

**Methods:**

The review process was iterative due to our goal to inductively develop the identified constructs. Data collection occurred in four primary stages: (1) an initial set of key literature across disciplines was identified and used to inform (2) journal and (3) author searches that, in turn, informed the design of the final (4) database search. Additionally, (5) we conducted citation searches of relevant literature reviews identified in each stage. We carried out an inductive thematic content analysis with the goal of developing homogenous, well-defined, and mutually exclusive categories.

**Results:**

We identified eight external context constructs: (1) professional influences, (2) political support, (3) social climate, (4) local infrastructure, (5) policy and legal climate, (6) relational climate, (7) target population, and (8) funding and economic climate.

**Conclusions:**

This is the first study to our knowledge to use a systematic review process to identify empirically observed external context factors documented to impact implementation. Comparison with four widely-utilized implementation frameworks supports the exhaustiveness of our review process. Future work should focus on the development of more stringent operationalization and measurement of these external constructs.

**Electronic supplementary material:**

The online version of this article (10.1186/s12913-018-3046-5) contains supplementary material, which is available to authorized users.

## Background

Considerable scientific effort has focused on identifying factors affecting translation of evidence-based interventions (EBIs) from research to practice. While conceptual models and empirical studies emphasize the importance of the external implementation context—i.e., factors existing outside the boundaries of the entity or entities leading the implementation of one or more EBIs—to the translation process, numerous factors point to limitations of its current conceptualization, including the absence of a comprehensive external context measure and its constituent factors. Moreover, current frameworks that generally define the external context are rooted in theoretical conceptualizations, rather than observed instances of external factors affecting implementation. The current paper highlights limitations of extant conceptualizations of the external implementation setting and then describes a integrative systematic literature review aimed at developing an inductive conceptualization of the external context.

While there is an extensive body of literature focused on the identification of facilitators and barriers organizations encounter when implementing EBIs [1, 2], empirical research in this area has focused largely on influences internal to implementing organizations. This is a considerable gap considering the recognized influence of both internal and external factors to the implementation process and because external factors are often antecedents of organizational readiness, drive organizational-level policy and processes, and pose greater difficulties in addressing than internal factors because they are typically beyond any single organization’s control to easily change [3–9]. Instrument reviews by both Clinton-McHarg et al. [10] and Lewis et al. [11] have demonstrated lack of consideration of the external context among validated implementation measures. While some instruments identified in these reviews measure selected aspects of the external context, no one instrument identified focuses explicitly on it. This is problematic because it leads researchers to one or a combination of the following options when seeking to understand the impact of the external context on the implementation process: (1) combine single items from multiple instruments; (2) use each identified instrument in its entirety; or (3) create untested, home-grown measures. All of these options cause obvious problems related to consistency, replicability, and comparability across implementation studies [11].

While defined within some existing implementation frameworks (e.g., [3–6]), there are inconsistencies regarding what these theoretical guides consider the external context to comprise. For instance, while some frameworks include social climate factors related to the larger community within which the intervention is embedded (e.g., [5]), others do not (e.g., [3, 4]). Moreover, frameworks sometimes include important constructs but do not provide detailed operationalizations for them (e.g., [5, 6]). Apart from existing implementation frameworks, Birken et al. [8] have pointed to organizational theory as an area where researchers can look to better understand the external context. They specifically highlight how “[o]rganizational theories describe, explain, and predict the complex interaction between organizations and their external environments” (p. 2) and how these interactions influence organizational decisions and behavior [12–15]. While useful, organizational theories are not implementation-specific and likely lack important insights necessary for understanding the noted impact of the broader implementation context on different aspects or stages of the implementation process [16]. Given the above stated issues, a stronger and more consistent operationalization of the external context would greatly benefit the field.

More well-defined definitions for the external context and its constituent parts would be particularly beneficial for studies of complex interventions made of multiple interrelating components that extend into and interact with larger systems and communities within which they are embedded [17–20]. As an example, Housing First, a model for serving chronically homeless individuals with serious mental illness and substance use disorder [13], is a highly complex EBI because it requires interaction between multiple individuals (e.g., providers, case managers, landlords), organizations (e.g., government funders, non-profit service providers, property management) and systems (e.g., housing, medical, mental health, substance abuse) to be successful [19, 21–23]. As such, it requires significant relational coordination with external entities. Previous research has also demonstrated how external factors such as community stigma and broader politics often result in 'not in my back yard' attitudes that can negatively impact model implementation [24, 25]. While external factors such as these are captured to some extent through concepts like 'cosmopolitanism' [3], 'interorganizational networks' [4, 6], 'interorganizational relationships' [6], 'sociopolitical' [4], 'social climate' [5], and 'political climate' [5] found in existing implementation frameworks, no one framework captures them all.

Our goal in the current literature review was to identify a more exhaustive list of external context factors impacting the implementation of complex interventions than what is explicated in current models and frameworks produced through synthesis of pre-existing theory [3–6, 26]. We employed an integrative review process that aimed to indictively develop a taxonomy of external context constructs  based in empirical observations existing in the identified literature.

## Methods

We conducted an integrative literature review because of its usefulness for generating theory and classifications of constructs [27–29]. The standard process for an integrative review includes the following steps: (1) problem formulation, (2) data collection, (3) evaluation of data, (4) data analysis, and (5) interpretation and presentation of the results [29]. The *primary problem* motivating this review was the lack of a tool to measure the external implementation context for complex interventions. As such, our primary research question was: What external context factors have been demonstrated to impact the implementation of complex health interventions (and social service interventions with health implications) within the empirical literature? Because of the appropriateness of inductive processes for theory and construct development, we conducted an iterative literature review where sampling at each stage was informed by literature identified in prior stages [27, 30].

### Data collection process (July 2014–July 2015)

To be included for initial screening, articles were required to: (1) be written in English; (2) describe empirically observed external context factors (i.e., facilitators or barriers existing outside the boundaries of a particular organization or organizations implementing the intervention) affecting the implementation of a complex intervention or interventions, i.e., “interventions that contain several interacting components” [18] (p. 1); and (3) describe an intervention with impact (or reasonably ascertainable impact) on client- or population-level outcomes. We excluded any article that (4) only discussed external context factors as theoretical barriers or facilitators or (5) focused on interventions we understood to only impact organizational- or staff-level outcomes. Qualitative, quantitative, and mixed method articles were all included. Additionally, if a review article was encountered at any stage in the search process, we screened the original articles discussed within the review for inclusion into our analysis. All identified articles were processed using Zotero bibliographic management software [31]. When an article met basic inclusion criteria or when there was not enough information to tell from the title or abstract, we loaded the article into MAXQDA qualitative analysis software for further review and potential coding [32].

**Fig. 1:**
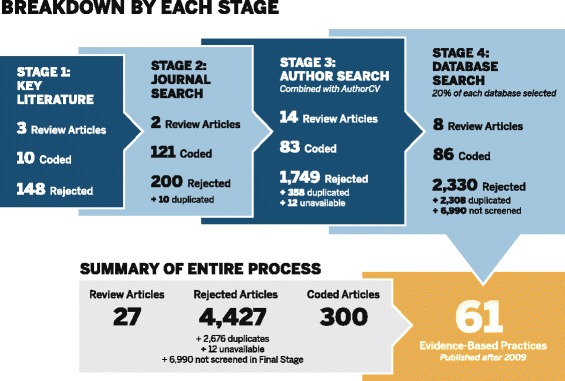
Flow diagram with detailed overview of the literature identification and screening process. Inclusion criteria for search: (1) written in English; (2) describe empirically observed external context factors affecting the implementation of a complex intervention or interventions; and (3) describe an intervention with impact on client- or population-level outcomes. Search exclusion criteria: (1) discussed external context factors as theoretical barriers or facilitators; (2) focused on interventions we understood to only impact organizational- or staff-level outcomes

We collected data in four stages: (1) identification of known literature, (2) journal search, (3) author search, and (4) database search (Fig. 1 presents an overview of this process). These stages were identified after consultation with our university’s public health librarian (HC), who developed a strategy to specifically start with a review of the recent literature narrowly defined by our initial problem definition and expand to be more expansive in scope with each stage. (Engagement of librarians in systematic review design is demonstrated to lead to more comprehensive search strategies utilizing more exhaustive techniques than typically followed [33].)

The need for a multi-stage process was evidenced by the librarian’s initial searches that suggested the literature was too widely dispersed across disciplines for a keyword search to be effective. As such, the iterative process developed was aimed at identifying different strands of literature using different terminology to express similar concepts, which could inform a final database search (e.g., journal and author specific keywords and time parameters). Therefore, our search followed a logic similar to that of snowball sampling frequently used in qualitative research [34]. Reflecting our iterative approach, coding and category development started in Stage 1 and continued throughout the data collection process.

The goal of **Stage 1** (July 2014 through November, 2014) was to identify articles discussing issues related to implementation in the external context to serve as a foundation for the search. To accomplish this, the Principal Investigator (DPW) first identified 22 documents discussing external context issues in Housing First programming (an intervention he has expert knowledge of) and articles discussing more general issues related to the external implementation context that he was already familiar with. Three of these articles were literature reviews, from which we identified an additional 139 articles (*n* = 161). Only 10 of these articles met inclusion criteria to continue to the next stage.

The goal of **Stage 2** (November 2014) was to identify communities studying and conversing about the implementation of complex interventions using articles identified in Stage 1 as a guide. We identified 16 journals likely to publish literature on the external implementation context from the Stage 1 key literature and DPW’s expert knowledge of the topic. Because our list of journals was interdisciplinary and terminology is often discipline specific, HC created a list of search terms specific to each journal based on keywords from articles published within them that were identified in Stage 1 (see Additional file 1). To keep the search narrow at this stage, we randomly chose to search issues published in the years 2008, 2009, 2012, and 2013. For those journals without keywords, we manually searched the table of contents.

In **Stage 3** (December 2015 through January 2015), we identified the 25 most relevant authors for our search based on the frequency of their publications in Stage 1 and the relevance of their work to the review (see Additional file 1). Only first and senior authors of articles were considered (as they were most likely of all included authors to have a strong body of research related to the goals of the review). We next identified the complete publication history for selected authors using Scopus (Elsevier citation database) and a search of their curriculum vitaes, which assisted us in our final refinement of parameters for Stage 4 (i.e., the database search).

In **Stage 4** (July 2015),^1^ we used database-specific controlled vocabulary and keywords used by authors identified in the previous stages to design the database search. We ran a series of searches in the following databases: PubMed, PsychINFO, CINAHL, and Academic Search Premier. We used the previously generated keywords and phrases to identify appropriate controlled vocabulary for each database (see Additional file 1). Both controlled vocabulary terms and keywords or phrases were combined with filters (e.g., English language, publication date, peer-reviewed articles) to focus the search results. A second search of PubMed was also conducted using the specific name of the At Home/Chez Soi Housing First project, as we were aware individuals studying the program were publishing a considerable amount of new implementation literature on this EBI at the time, which we wanted to ensure we captured since previously used search terms were not picking it up. As we had already coded articles from previous stages and were approaching saturation of themes, we decided to limit the number of the database results we would review. We first separated these into one of four categories based on the primary discipline of the journal they were published in (e.g., medicine, public health, interdisciplinary, other). We then randomly selected 20% of the articles in each category to be coded. If duplicate articles from previous stages or foreign language articles were found, we replaced the article with another randomly-selected one from the same category and database. Additionally, to avoid redundancy and oversaturation, we did not code any articles for which we had already identified five or more articles from that author in earlier stages.

A detailed flow chart outlining the article screening process is depicted in Fig. 1 (a simplified PRISMA flow diagram is included in Additional file 2). Through this entire process, we identified a total of 14,432 articles. Of these, 2676 were duplicate articles, 4427 (including those marked as non-empirical and foreign language) were rejected for not meeting inclusion criteria, 27 were review articles that helped us identify original works, and 12 did not have full text versions we could locate. There were an additional 6990 articles identified in Stage 4 that we did not screen because we were reaching saturation of themes at that point (see Data Analysis section below). This left a total of 217 unique articles, which were fully coded. Due to this large number of articles, we have chosen to focus only on 61 describing EBIs published after 2009 because: (1) there is a potential difference in factors affecting implementation of interventions demonstrated to be effective (i.e., EBIs) and (2) it is reasonable to expect attention to external factors would be more salient after 2009 because this is when the first article describing the Consolidated Framework for Implementation Research (CFIR), an implementation framework that includes an 'outter setting' domain, was published, and this framework has had considerable influence over the operationalization of constructs affecting the implementation process. Due to heterogeneity of criteria depending on discipline of origin (i.e., medicine, behavioral health, public health, criminal justice), we defined an intervention as an EBI if: (1) the article stated it was an EBI or that there was prior evidence of its efficacy/effectiveness; (2) we were familiar with the intervention as an EBI; or (3) we were able to find evidence of its status as an EBI through an online search.

### Data analysis

We conducted an inductive thematic content analysis with the goal of establishing homogenous, well-defined, and mutually exclusive categories from which to develop our constructs [27, 35–37]. First, two researchers (DPW and ELA), developed a list of preliminary codes after reviewing the articles identified in Stage 1. Next, three research assistants (JC, JJ, and IO) and ELA coded instances where external context factors were discussed in the 217 unique articles (see above) using our preliminary codes. When a coder encountered a facilitator or barrier not fitting the initial list, we discussed it as a group and developed a new code if warranted. Approximately every 2 months during the process, we used 10% of the articles to establish interrater agreement by looking at the degree of overlap in codes in MAXQDA [38]. We did not move coding forward until interrater agreement was established at 80%. *Evaluation of the data* was conducted during the coding process: only one article was identified that did not appropriately explain external context factors to warrant its inclusion in the review.

DPW conducted a second round of analysis in which he reviewed segments coded by the other researchers to develop more exact and thorough categories, focusing on the 61 EBI articles published after 2009. This activity also served as a quality check on the previous round of coding, and all instances where he identified a passage of text that may have been inappropriately coded were discussed as a team and recoded if discussion warranted. This process resulted in the development of eight overarching constructs representing the external context, which are described in detail below.

Once coding was completed, we created a two-by-two matrix of all codes to ensure there was no substantial overlap between them that would require collapsing or redefining of categories. We also counted the number of articles each category appeared in to understand how strongly represented it was within the sample (we did not count the frequency of times the code appeared, as there was potential that multiple coding instances of the same category within a document might be reflective of a single issue).

## Results

Of the 61 articles included in the analysis, 43 [39–81] were research articles and 18 [82–99] were non-research articles (e.g., practice articles, policy updates, issue pieces, case studies, and commentaries). Additionally, 48 articles [39–41, 43, 44, 48–53, 55, 57–68, 71–80, 82, 83, 86–93, 96–99] discussed a single intervention; 6 [47, 54, 81, 84, 85, 94] discussed multiple interventions related to the same health problem; and 7 [42, 45, 46, 56, 69, 70, 95] discussed issues related to the implementation of interventions associated with a specific health problem more generally. Regarding the issues interventions sought to address: 27 [42–44, 47–50, 52, 54, 55, 58–61, 63, 68, 69, 73, 82, 86, 87, 91, 93–95, 97] discussed behavioral health, mental health, or substance use interventions; 16 [45, 51, 56, 57, 62, 64, 67, 72, 76, 79, 81, 85, 88, 89, 96, 99] discussed public health or prevention interventions; 7 [53, 65, 74, 75, 83, 90, 92] discussed homelessness interventions (with aspects having overlap with both public health and behavioral health); 6 [41, 46, 66, 71, 78, 80] discussed medical, primary, or integrated care interventions; 4 [39, 40, 77, 84] were interventions in the area of parenting and/or child welfare; and 1 [98] was a criminal justice intervention.

**Fig. 2:**
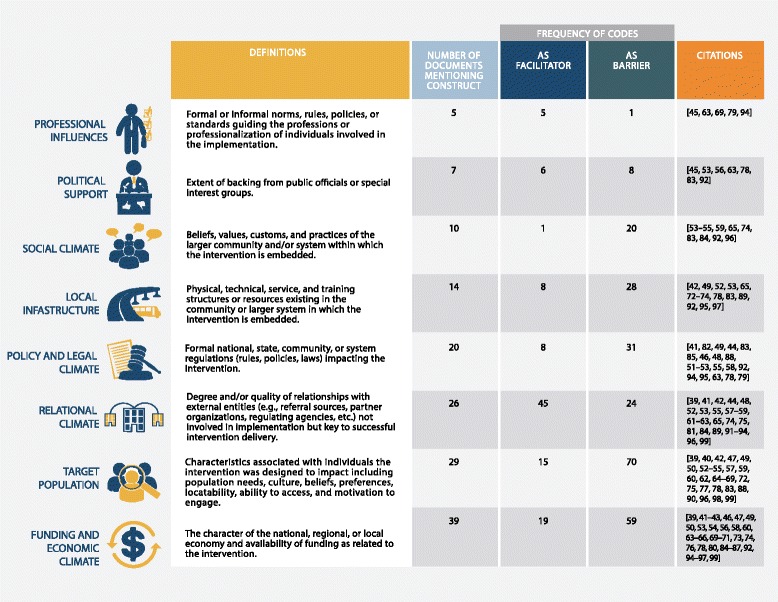
Taxonomy of external context constructs identified, their definitions, and frequency of coding in sample. Barrier and facilitator counts refer to the total number of times the issue was mentioned within the sample and do not consider the coding of multiple mentions of the same barrier or facilitator within a single article. Therefore, the document count is a better indicator of the extent to which the construct was discussed within the sample

We identified a total of eight constructs listed in Fig. 2. The table also includes the number of articles each construct was mentioned in and the number of times the construct was coded as a barrier or facilitator in these articles. We define each construct below and provide examples from the sampled articles.

### Professional influences

Five articles identified **professional influences** as impacting EBI implementation. We define professional influences as **formal or informal norms, rules, policies, or standards guiding the profession or professionalization of individuals involved in the implementation**. In their study of Assertive Community Treatment and Motivational Interviewing, Mcgraw et al. [94] provide an example of *informal professional influences* as a barrier to implementation when they discuss difficulties recruiting psychiatrists willing to work in a community setting because the work requires a “different mindset” than most psychiatrists have and it “takes a long time to find [a psychiatrist] willing to work outside of their comfort zone” (p. 202). Winickoff et al. [79] provide the only example of a *formal professional influence* when they discuss how an amendment to an American Medical Association policy recommending “clinicians treat people who smoke with the available tobacco dependence treatments regardless of the clinical context” (p. 114) facilitated the implementation of a nicotine replacement therapy intervention.

### Political support

**Political support**, identified in 7 articles, refers to the **extent of backing from public officials or special interest groups** (e.g., lobbyists or representatives of an occupational group). Political actors were demonstrated to have either a negative or positive influence on implementation depending on whether they were in *support or opposition* to the intervention(s) in question. For instance, Menear et al. [63] described how advocacy for supported employment by “academics and foreign experts” (p. 1034) increased the intervention’s professionalization (e.g., the process a trade or occupation goes through to become a true profession), thus having a positive impact on implementation. Demonstrating how political support can act as a barrier, Knutagard and Kristiansen [92] described how support for traditional housing services by public figures and organizations frustrated implementation of Housing First programming in Sweden: “In some cases, we were told that municipal representatives thought that Housing First would compete with their existing services, which they believed worked in a satisfactory manner” (p. 103).

### Social climate

The **social climate** refers to **beliefs, values, customs, and practices of the larger community or system** within which the intervention is set. Ten articles identified issues affecting implementation that were part of the social environment. Benjaminson [83] and Nelson et al. [65] both noted how a *commitment to traditional treatment models* within the local communities they studied led to difficulties implementing Housing First programming because it resulted in resistance to the intervention's underlying paradigm. *Negative attitudes and stigma* toward particular groups (e.g., minorities, people with mental illness, ex-offenders) were also demonstrated implementation barriers. Such was the case with Hasson et al.’s [55] study in which they reported “s[k]epticism…about people with mental illness working” (p. 339) to be a barrier to the implementation of supported employment in Sweden. Demonstrating how social climate factors related to the larger systems within which an intervention is embedded, Chamberlain et al. [84] discussed how conflict arose when implementing an American-designed child welfare intervention in England due to “cultural differences” (p. 282) that impacted attitudes toward evidence-based practices.

### Local infrastructure

Fourteen documents discussed the **local infrastructure**, i.e., **physical, technical, or service structures or resources**, in the larger service system or community as impacting EBI implementation. Both Glisson et al. [52] and Nelson et al. [65] identified lack of local transportation as problematic for implementation of the interventions they respectively studied. Glisson et al. noted how lack of public transportation interfered with clients’ ability to access supportive services, while Nelson et al. discussed how it led to social isolation among clients who did not have alternate modes of transportation to interact with family and friends. In Glisson’s case, implementation was facilitated after the program took measures to address lack of public transportation in the community. A number of housing interventions discussed the importance of having available housing in the community as being integral to successful implementation [53, 65, 74, 83, 92]. Similarly, Schneider and Akhtar [97] noted lack of available jobs in the community to be a barrier to implementation of supported employment. Amodeo et al. [42] point to both system and community barriers to the implementation of substance abuse treatment services:

…the theme of “lack of concrete services” emerged, with respondent comments including the following: for homeless population, lack of housing is a barrier,” “for co-occurring psychiatric and physical disorders, very limited resources,” and “lack of resources in the county (no bus system, few jobs),” and “project had limited access to substance abuse treatment. (p. 386)

Lack of infrastructure and resources for training were noted to stall implementation. For instance, “[l]ack availability of training facilities” (p. 1301) within the larger service system was a noted barrier in van Bodegom-Vos et al.’s [78] study of a rheumatoid arthritis intervention, and Hyder et al.’s [89] study of a road traffic safety intervention identified lack of local capacity for providing training and technical assistance as a problem.

### Policy and legal climate

The **policy and legal climate**, referring to **external regulations in the form of rules, policies, and laws***,* demonstrated to impact EBI implementation were discussed in 20 articles. *Overly complicated, strict, burdensome, and unclear policies*, including “unnecessary red tape within service systems” [52] (p. 3), were demonstrated to interfere with implementation of both mental health and medical interventions [41, 49, 52, 78, 82]. Some policies were demonstrated to specifically prevent funding of services key to the intervention, as in the case of Collins et al.’s study of an HIV intervention that was prevented from receiving U.S. Centers for Disease Control and Prevention funding due to policies within the government organization. Alexander et al.’s study demonstrated how “misalignment between current payment systems [rules] and [patient-centered medical home] goals” (p. 149) interfered with implementation of patient-centered medical homes.

*Conflicting/competing demands* related to polices of different government agencies and/or multiple program funders were a noted barrier, as in the case of McGraw et al.’s [94] study when they state “competing demands of multiple funding sources and the requirement to collaborate with local agencies…complicated the implementation” (p. 208) of EBIs for mental health. In the case of supported employment, policies were demonstrated to impair intervention acceptability for supported employment clients when obtaining a job could lead to a loss of benefits they considered to be important [44, 55].

In some cases, the policy and legal context was demonstrated to be a *coercive external force* that could facilitate EBI implementation. One such example was in Greenwood et al.’s [53] study where the “legal duty [of]…local authorities to rehouse all homeless people” (p. 308) helped facilitate implementation of a Scottish Housing First program. In a second example, employment authorities applied sufficient pressure through contracts and funding schemes to move several employment agencies toward a supported employment model [63].

### Relational climate

Twenty-six articles discussed the external **relational climate**, or the **degree and/or quality of relationships with external entities (e.g., referral sources, partner organizations, regulating agencies, etc.) not involved in implementation but key to successful intervention delivery**, as impacting implementation. Various aspects of the relational climate were discussed. Having *buy-in* or *support* from influential organizations was demonstrated to have a positive impact on implementation, as in the case of Lloyd et al.’s [93] study of Foundations of Learning, where gaining support of Head Start agencies was a noted facilitator. *Lack of buy-in* from homeless service providers who favored alternative, non-evidence-based approaches was demonstrated to be a barrier in Knutagard and Kristiansen’s [92] and Greenwood et al.’s [53] studies of Housing First programming.

Strong *partnerships* with outside entities were demonstrated to facilitate EBI implementation, while poor or tarnished relationships with partners were a demonstrated barrier. For instance, in their study of Housing First, Nelson et al. [65] pointed to “partnerships with government agencies and departments [enhancing] the project’s ability to secure access to housing”, while tarnished relationships with landlords led to loss of housing options as they chose to leave the program. Likewise, Robinson et al. [96] demonstrate the importance of partnerships for providing referral and recruitment opportunities when they state “[p]artnering with stakeholders…was important in helping to overcome agency deficiencies in their connections with the [target] community” (p. 215) in their study of Community PROMISE for HIV.

### Target population

Factors associated with the intervention’s **target population**, i.e., **those individuals the intervention was designed to serve or impact**, were discussed in 30 articles, with the *needs* of the target population being the most mentioned issue related to this topic. Needs related to reading comprehension [67], developmental stage [67, 83], transportation [42, 67], mental health [59, 98], finances [83], and scheduling (largely related to work and childcare) [40, 62, 88] were all demonstrated to negatively impact members of the target population’s capacity to engage in a wide range of interventions. Parker et al. [67] provide an example of needs related to developmental stage in their study of a Positive Prevention program adapted for youth living with HIV/AIDS where “the study team was not prepared for the degree to which the youth were delayed in their ability to read and write, potentially as a result of cognitive development deficiencies due to HIV” (p. 144).

The target population’s *ability to access* the intervention was demonstrated to be important. Intervention access had overlap with scheduling needs, as conflicts with work, school, or public transportation schedules were all noted implementation barriers. Such was the case with Perlick et al.’s [68] study of multifamily group treatment in which a number of veterans refused participation due to “work- or school-related scheduling conflict or feeling too busy” (p. 536). Lack of sufficient health insurance and social benefits were also demonstrated implementation barriers, as in the case of El-Mallakh et al.’s [49] study of MedMAP, a psychotropic medication management intervention, in which participants “were unable to afford costly medications” (p. 521) and Benjaminson et al.’s [83] discussion of Housing First, in which they noted lack of cash benefits as a barrier to finding affordable housing for young adults.

The *culture* of the target population was demonstrated to be important, as in the case of Robinson et al.’s [96] study of Community PROMISE. Robinson and colleagues noted how the location of the program for African American clients (whose culture is stigmatizing of homosexuality) in an area known to provide services to the gay community “presented a barrier for some [clients] who did not identify as gay or bisexual or were not open about their sexual experiences” (p. 215). Additionally, Stergiopoulos et al. [75] noted how language, an aspect of culture, was a barrier during the implementation of a Housing First program serving diverse group of clients.

Several factors related to the target population’s *motivation to engage* with the intervention were identified. For instance, Fox et al. [50] noted that a number of clients did “not see the value of participating in short-term follow-up evaluation” (p. 608) as part of the behavioral health intervention known as Parenting Young Children. Other issues mentioned that could negatively impact motivation of the target population included stigma [62], mistrust of the system an intervention is embedded in [53], and feeling as though they did not have enough time to participate fully [68]. Related to and possibly underlying motivation in some instances, the *preferences and beliefs* of the target population were also demonstrated to be important. Target population preferences were a facilitator for Benjaminson et al.’s [83] study of a Housing First intervention that found youth participants preferred temporary accommodations provided by the program to “emergency shelters or random couch surfing” (p. 126). Hasson et al. [55] demonstrated how target population members' beliefs that intervention participation might lead to discontinuation of government benefits or that they were unprepared/incapable of work in a competitive marketplace prevented some of them from participating in supported employment.

Finally, an important barrier associated the target population was when individuals in that population were *not available or difficult to locate*. An interesting example of this was in Silva et al.’s [72] study of a breast cancer screening intervention being implemented in Brazil, which had difficulty locating patients due to the transient nature of migrant worker lives. In the case of some youth interventions, difficulty locating parents could negatively impact the ability to engage the target population, which was the case in Langley et al.’s [60] study of Cognitive Behavioral Intervention for Trauma in Schools.

### Funding and economic climate

Discussed within 35 articles, aspects of the **funding and economic climate**, i.e., **the character of the national, regional, or local economy and availability of funding**, were the most frequently mentioned issue affecting intervention implementation. Issues with the *labor market* such as lack of skilled and experienced workers, high cost of skilled workers when they were able to be located, and high turnover were all noted barriers to implementation. In one example, Alexander et al. [41] discuss difficulties with staffing that arose during implementation of a patient-centered medical home intervention: “availability of primary care physicians was a major threat…because of increasing differentials in income and working conditions, fewer medical students were opting to go into primary care.” (p. 150).

The *availability of a stable funding source* aligned with the intervention and organizational processes were important. For clinical interventions, “insufficient [numbers] of patients with reimbursement coverage” [73] (p. 10) was demonstrated to be problematic. Problems related to funding availability were also impacted by changes in policies or larger economic shifts: “The severity of the economic crisis has contributed several obstacles…as funding for mental health continues to decline, providers have to locate areas to cut.” [95] (p. 464). *Incentive and reimbursement structures* misaligned with the intervention were also demonstrated to negatively impact implementation. In the article by Sanchez et al. [71], the authors describe how reimbursement issues negatively impacted implementation of an integrated care program:

…financial issues present substantial obstacles to integrated health care…respondents predominately identified lack of reimbursement for clinical care management and paraprofessional services, followed by lack of reimbursement for screening services and consultation between primary care and behavioral health providers. (p. 31)

## Discussion

To our knowledge, this is the first study to use a systematic review of empirical literature to identify external context factors documented to impact EBI implementation. While the large number of articles meeting our inclusion criteria suggest there has been a focus on the external context within the literature, the reality is that the majority of external context findings studies identified were the result of passive or exploratory endeavors, rather than purposeful research questions seeking to understand external context factors.

As demonstrated in Table 1, the external context constructs identified overlap somewhat, but not completely, with existing theoretical models and frameworks including: the CFIR [3], Exploration, Preparation, Implementation, Sustainment (EPIS) [4], Integrated Promoting Action on Research Implementation in Health Services (i-PARiHS; revised version of the original PARiHS) [6], and the Multi-level framework (MLF) predicting implementation outcomes [5]. We have chosen to focus on these frameworks in our discussion because they are highly cited and/or applied widely within the implementation literature.

**Table 1:** Comparison of constructs evidenced through literature review with external factor constructs in existing frameworks

	Consolidate Framework for Implementation Research (CFIR) [3]	Exploration, Preparation, Implementation, Sustainment (EPIS) [4]^a^	Integrated Promoting Action on Research Implementation in Health Services (i-PARiHS) [6]^b^	Multi-level framework (MLF) predicting implementation outcomes [5]
Professional influences	--	Interorganizational networks	--	--
Political support	--	Sociopolitical; Client advocacy	--	Political or social climate
Social climate	--	--	--	Political or social climate
Local infrastructure	--	--	--	Infrastructure
Policy & legal climate	External policies and incentives	--	Policy drivers & priorities; incentives & mandates; regulatory frameworks	Public policies
Relational climate	Cosmopolitanism	Interorganizational networks	Interorganizational networks & relationships	--
Target population	Patient needs and resources	Client advocacy	--	--
Economic & funding climate	--	Funding	--	Economic climate
No directly comparable construct or too broad to directly parallel to identified constructs	Peer pressure	Intervention developers; Leadership	Environmental (in) stability (definition unclear)	Physical environment

'--' = No directly comparable construct

^a^Only the active implementation phase of the EPIS framework is considered here since this was the focus of the current literature review

^b^We focus on the revised version of the PARiHS, as the original did not address the external context; The i-PARiHS is limited in its conceptualization of the external context, as it only considers the external health system

While not any one of these four frameworks fully encapsulates the constructs identified through our review, each construct is represented in one way or another when the frameworks are considered in combination. As such, using only one of these frameworks as a guide to understanding the external context raises the risk that a key aspect of it might be overlooked. One potential reason for the inconsistent representation of our constructs across the existing frameworks is the context of these frameworks’ developments. For instance, the CFIR, PARiHS, and MLF were developed to explain implementation in healthcare-specific settings, while EPIS was developed within the context of more social service-oriented programming [4, 5, 26, 100]. Furthermore, the revised i-PARiHS (for which the original version did not consider the external context [26]) is focused explicitly on the health system (as opposed to the broader community) as its external context [6]. Our approach, however, was broader in that it included multiple types of health-related interventions regardless of their setting (e.g., healthcare or social service).

Just as these individual frameworks did not completely capture our constructs, our review did not find evidence of all aspects of the outer context identified within them. While we did not come across any instances where external ‘peer pressure’, an aspect of the CFIR’s ‘outer setting’, was discussed as having an impact on implementation, it is important to note this is considered a substantial motivator for the adoption of new practices within the organizational literature [13]. The reason the EPIS constructs ‘leadership' and ‘intervention developers’ did not stand out as themes within our analysis is because we largely considered these to be aspects of the internal context. This is because (even if they are originally external to the organization) we understand their involvement with the implementation process to place them in a role that can be considered part of the organization. It is important to note the CFIR also does not consider leadership and intervention developers to be part of the external context, placing them instead in its ‘inner setting’ and ‘intervention characteristics’ domains respectively [101].

Additionally, the physical environment, which is part of the MLF that includes such aspects as weather, topography, and the condition of the built human environment [5], was not evidenced in our review. While distance between the intervention and the individual was sometimes cited as impacting access, this was discussed more as an issue having to do with transportation than the physical environment. It is possible the physical environment would have stood out as a construct in its own right if we had not restricted our review to interventions we were able to identify as EBIs. For instance, an article by Zarrett et al. [102], which was excluded from our review, discussed how weather variations posed problems for a school-based physical activity intervention that was largely facilitated outdoors. Likewise, Colon et al., [103] discussed quality of sidewalks as a barrier to a walking intervention targeting underserved African American communities. Restricting our review to EBIs also excluded a number of articles looking at interventions in developing countries where the physical environment may have played a more salient role during the implementation process. Broadening our criteria to look at other phases of implementation may have also impacted our results. For instance, the literature on scaling of interventions tends to more robustly consider the implementation context due to the need to develop partnerships and engage political support [104]. For instance, a synthesis of models and frameworks for scaling of public health interventions conducted by Milat et al. [105] identified active engagement of community members and political will as important elements of the scaling-up process. The lack of explicit focus on the external context within the literature impacted the quality of the data available for our analysis, as it often resulted in incomplete explanations through which external context factors impacted implementation. This was particularly the case with the quantitative studies in our sample, which would often list an external context factor such as 'policies' or 'funding' without any additional context or explanation.

Another difficulty we encountered during the analysis process was clearly defining the often-fuzzy boundaries between the external and internal context. As Damschroder et al. [3] note:

…the line between inner and outer setting is not always clear and the interface is dynamic and sometimes precarious. The specific factors considered ‘in’ or ‘out’ will depend on the context of the implementation effort. For example, outlying clinics may be part of the outer setting in one study, but part of the inner setting in another study. (p. 5)

Therefore, we had to make subjective value judgments as to where these boundaries lie given the information available within the article. Additionally, while we do point to the frequency of times a construct was mentioned as a facilitator or barrier, it is difficult to predict in which instances a construct will act as one or the other due to the contextual factors influencing their particular effects on an intervention [106]. For instance, political support may be desired when it is received from well-regarded individuals and better avoided when it comes from unpopular sources.

Despite our goal of establishing completely mutually exclusive categories, there is some minor overlap between constructs that could not be avoided. For instance, lack of public transportation is both a ‘local infrastructure’ issue and it creates a need for transportation assistance within the ‘target population’. Issues related to funding were noted within both the ‘policy and legal climate’ and ‘economic and funding climate’, as policies often dictate what funding can be used for and create cumbersome processes attached to it. Furthermore, the ‘policy and legal climate’ can at times have direct impacts on ‘professional influences’ through regulations and ‘local infrastructure’ by prioritizing or de-emphasizing resources for development or sustainability. Finally, stigma existing in the larger ‘social climate’ can be internalized by members of the ‘target population’, thus leading to perceptions and beliefs that can impact acceptability of an intervention [107].

In addition to the above noted challenges, our choice to limit the timespan of articles analyzed for this paper to post-CFIR literature and to exclude gray literature may have also limited our results. However, degree of saturation in our themes (e.g., the extent to which adding new articles yielded no new information) and the extent of overlap they have with the frameworks considered in Table 1 does support the exhaustiveness of our review process [34]. Our choice to conduct a review of empirical literature also has strengths over the development of similar constructs, as the well-known and previously discussed frameworks were developed based on reviews of other models and theories [3–5], which may not have been based in empirical evidence themselves. Due to limitations in the studies reviewed, we were unable to determine any causal links between constructs and outcomes. Furthermore, our findings do not preclude the existence of other factors which may have impacted outcomes in these or other studies. The link between specific contextual factors and outcomes is one that is yet to be established in implementation science and is of utmost importance when seeking to develop and test effective implementation strategies [108]. It is our hope that the findings presented in this paper will be a first step in developing stronger operationalizations necessary to establish the impact of external context factors on implementaiton outcomes. Finally, it is possible our iterative approach may have failed to capture some relevant articles. Though, we do not consider this problematic considering our goal was the development of comprehensive themes through qualitative saturation, rather than an exhaustive identification of articles.

## Conclusion

In summary, we identified eight constructs representing the external context through our integrative systematic review process. This list of constructs was more exhaustive than those proposed in any single one of the four implementation frameworks we compared them to. The incomplete representation of the external context within these frameworks is not meant to invalidate the usefulness of pre-existing theory. Indeed, existing implementation frameworks are incredibly useful guides that the external context constructs identified in this paper might be used in combination with depending on the needs of the individual study. Future work should seek to *further operationalize* the constructs identified for stronger measurement of the external context in an effort to better understand *how and to what degree* they impact the implementation process. Finally, additional work focusing on the external context as it relates to implementation of non-EBIs and low-resource settings has potential to evidence additional constructs.

## Additional files

Additional file 1: Detailed search information related to specific stages. This file includes tables containing detailed information related to Stage 2, Stage 3, and Stage 4 of the search. (PDF 253 kb)

Additional file 2: PRISMA 2009 flow diagram. This file contains a flow chart conforming to PRISMA guidelines that describes article screening process. (PDF 226 kb)

## Abbreviations

CFIR: Consolidated Framework for Implementation Research
EBI: Evidence-based intervention
EPIS: Exploration, Preparation, Implementation, Sustainment framework
MLF: Multi-level Framework Predicting Implementation Outcomes

## Footnotes

1 There was a large gap in time between this and the previous data collection stage because we were conducting preliminary analysis to inform our next steps.
