# Supplementary material for: Defining the external implementation context: an integrative systematic literature review
Source: BMC Health Serv Res. 2018 Mar 27;18:209. doi: 10.1186/s12913-018-3046-5 (PMC5870506; doi:10.1186/s12913-018-3046-5)
Supplement: Supplementary file 1 — Detailed search information related to specific stages. This file includes tables containing detailed information related to Stage 2, Stage 3, and Stage 4 of the search. (PDF 253 kb) [file 12913_2018_3046_MOESM1_ESM.pdf]

Stage 2 journals and journal-specific search terms/logic 2008, 2009, 2012, 2013			
	Journal Name	Journal-specific search terms	Type of search
1	Academy of Management Review	complexity, complex interventions, emergence, evidence-based practice(s),fidelity, harm reduction, health innovation, Housing First, Implementation (research), mental health services, organizational change, policy, policy transfer, readiness for change, realism, science and service, state mental health authority, supportive housing, translation two research	Manual search of journal's table of contents
2	Administration and Policy in Mental Health and Mental Health Services Research	Implementation, evidence based practice, mental health services, translation two research, science and service	Database search in Proquest Public Health
3	AIDS Education and Research	complexity, complex interventions, emergence, evidence-based practice(s),fidelity, harm reduction, health innovation, Housing First, Implementation (research), mental health services, organizational change, policy, policy transfer, readiness for change, realism, science and service, state mental health authority, supportive housing, translation two research	Manual search of journal's table of contents
4	American Journal of Psychiatric Rehabilitation	Housing first, fidelity, supportive housing	Manual search of journal's table of contents
5	American Journal of Public Health	complexity, complex interventions, emergence, evidence-based practice(s),fidelity, harm reduction, health innovation, Housing First, Implementation (research), mental health services, organizational change, policy, policy transfer, readiness for change, realism, science and service, state mental health authority, supportive housing, translation two research	Manual search of journal's table of contents
6	Community Mental Health Journal	Evidence-based practices, implementation, state mental health authority, policy	Database search of SpringerLink
7	European Journal of Homelessness	Housing First; fidelity; policy transfer	Manual search of journal's table of contents
8	Implementation Science	Implementation, health innovation, evidence-based practice	Database search of Health Reference Center Academic

9	Issues in Mental Health Nursing	complexity, complex interventions, emergence, evidence-based practice(s),fidelity, harm reduction, health innovation, Housing First, Implementation (research), mental health services, organizational change, policy, policy transfer, readiness for change, realism, science and service, state mental health authority, supportive housing, translation two research	Manual search of journal's table of contents
10	Journal of Behavioral Health Services Research	complexity, complex interventions, emergence, evidence-based practice(s),fidelity, harm reduction, health innovation, Housing First, Implementation (research), mental health services, organizational change, policy, policy transfer, readiness for change, realism, science and service, state mental health authority, supportive housing, translation two research	Manual search of journal's table of contents
11	Journal of Dual Diagnosis	complexity, complex interventions, emergence, evidence-based practice(s),fidelity, harm reduction, health innovation, Housing First, Implementation (research), mental health services, organizational change, policy, policy transfer, readiness for change, realism, science and service, state mental health authority, supportive housing, translation two research	Manual search of journal's table of contents
12	Medical Care Research and Review	organizational change, readiness for change	Database search of Sage Health Sciences full text collection
13	Psychiatric Services	complexity, complex interventions, emergence, evidence-based practice(s),fidelity, harm reduction, health innovation, Housing First, Implementation (research), mental health services, organizational change, policy, policy transfer, readiness for change, realism, science and service, state mental health authority, supportive housing, translation two research	Manual search of journal's table of contents
14	Schizophrenia Bulletin	evidence-based practices, implementation research	Database search of PubMedCentral &<br>Search of journals website
15	Social Science and Medicine	Complexity, complex interventions, emergence, realism	Database search of ScienceDirect Journals

16	Substance Abuse Treatment, Prevention, and Policy	fidelity, implementation, harm reduction	Database search of BioMedCentral Open Access

Stage 2-identified authors and name variations published under		
Author name		Name variation
1	Broner, Nahama	n/a
2	Collins, Charles	Charles B. Collins, Jr
3	Greenwood, Ronni Michelle	n/a
4	Hofstede, Stefanie N.	n/a
5	McHugo, Greg	Gregory J. McHugo
6	Panzano, Phyllis C.	n/a
7	Aarons, Gregory A.	n/a
8	Bjorklund, Robert W.	n/a
9	Bond, Gary R.	n/a
10	Coulon, Sandra M.	n/a
11	Dadich, Ann	n/a
12	El-Mallakh, Peggy	n/a
13	Stefancic, Ana	n/a
14	van Bodegom-Vos, Leti	n/a
15	Robinson, Beatrice	Bean E. Robinson
16	Roth, Dee M.	n/a
17	Wilson, Dawn K.	n/a
18	Drake, Robert E.	n/a
19	Howard, Patricia B.	n/a
20	Ko, Nai-Ying	Ko, Naiying, Ko, Nai Ying
21	Ko, Wenchien	Ko, Wen-Chien, Ko, Wen Chien
22	Matoff-Stepp, Sabrina	n/a
23	Rapp, Charlie	Charles A. Rapp
24	Tsemberis, Sam	n/a
25	Whitley, Rob	n/a

**Stage 4 databases and database-specific search terms/logic**		
Database		Search terms used
1	PubMed (1st search)	"Health Plan Implementation"[MAJR]; "Health Plan Implementation/methods"[MAJR]; "Health Plan Implementation/organization and administration"[MAJR]; "Health Services Research/methods"[MAJR]; "Community Mental Health Services/methods"[MAJR]; "Community Mental Health Services/methods"[MAJR] AND "implement*"; "Health Services Research/methods"[MAJR] AND "implement*"; "Health Services Research"[MAJR] AND "implement*"
2	PubMed (2nd search)	"chez soi"; filter: Humans & Journal Article
3	PsycINFO	MM "Educational Program Planning" AND "implement*"; MM "Educational Program Evaluation" AND "implement*"; MM "Mental Health Program Evaluation" AND "implement*"; MM "Community Mental Health Services" AND "implement*"; MM "Program Development" AND "implement*"; "implementation 4trategy*" OR "implementation barrier*" OR "barriers to implementat*"; "implementation 4trategy*" OR "implementation barrier*" OR "barriers to implementat*" AND "intervention*
4	CINAHL	(MM "Program Implementation/ST"); (MM "Program Implementation/MT")
5	Academic Search Premier	DE "Program implementation (Education)"; DE "Implementation (Social action programs)"
