# Supplementary material for: Defining the external implementation context: an integrative systematic literature review
Source: BMC Health Serv Res. 2018 Mar 27;18:209. doi: 10.1186/s12913-018-3046-5 (PMC5870506; doi:10.1186/s12913-018-3046-5)
Supplement: Supplementary file 2 — PRISMA 2009 flow diagram. This file contains a flow chart conforming to PRISMA guidelines that describes article screening process. (PDF 226 kb) [file 12913_2018_3046_MOESM2_ESM.pdf]

PRISMA 2009 Flow Diagram

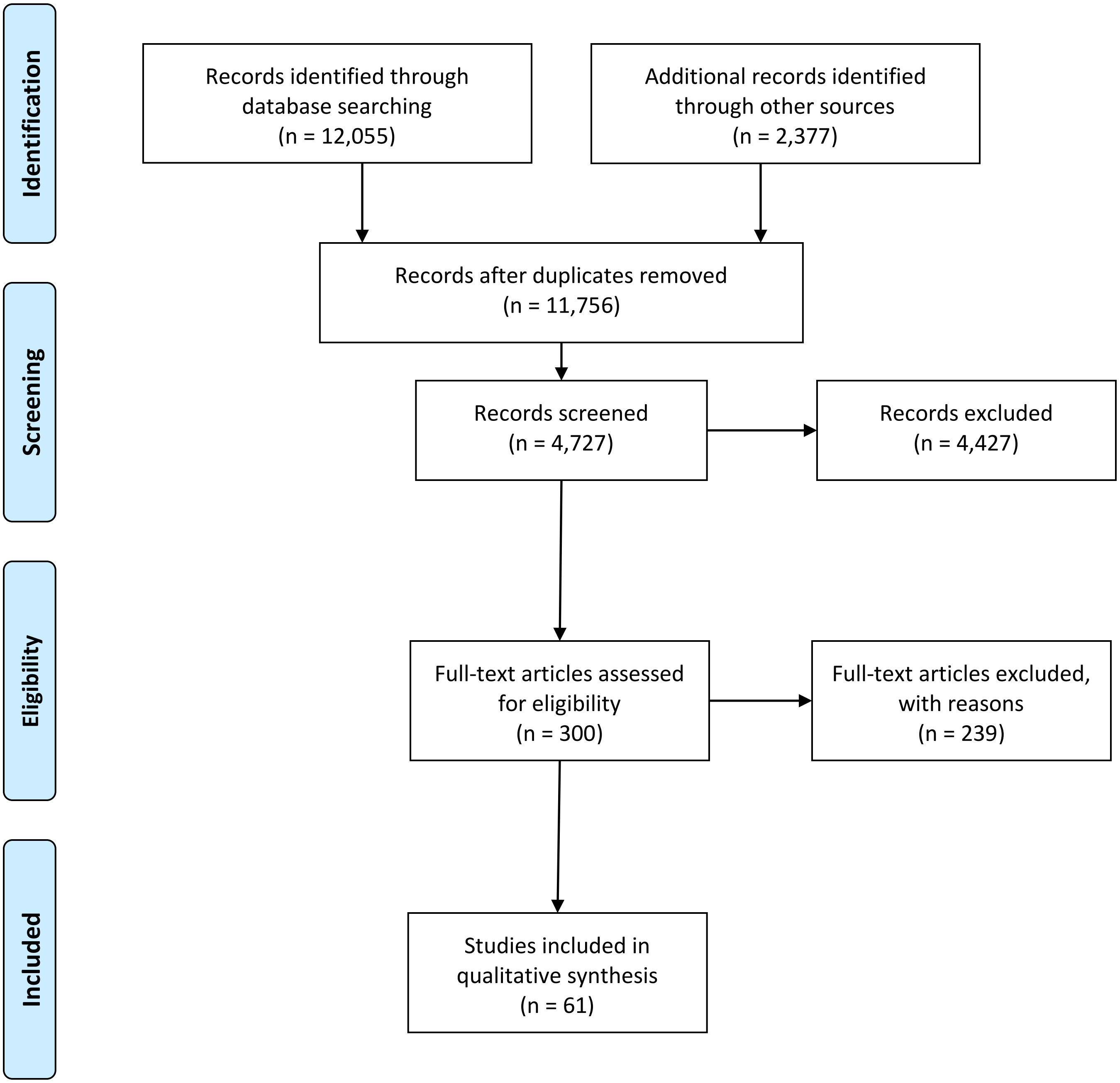

From: Moher D, Liberati A, Tetzlaff J, Altman DG, The PRISMA Group (2009). *P*referred *R*eporting *I*tems for *S*ystematic Reviews and *M*eta-*A*nalyses: The PRISMA Statement. PLoS Med 6(7): e1000097. doi:10.1371/journal.pmed1000097

For more information, visit www.prisma-statement.org.
